# Supplementary material for: Exercising healthy behaviors: A latent class analysis of positive coping during the COVID-19 pandemic and associations with alcohol-related and mental health outcomes
Source: PLoS One. 2024 Feb 14;19(2):e0297060. doi: 10.1371/journal.pone.0297060 (PMC10866465; doi:10.1371/journal.pone.0297060)
Supplement: S1 Table — (DOCX) [file pone.0297060.s001.docx]

**Supporting Information**

S1 Table. Full Results from Multiple Regression Models with Covariates and Coping by AUD Interaction Terms

|  | AUDIT (*n* = 463) | | |  | PSS (*n* = 463) | | |  | GAD-7 (*n* = 463) | | |
| --- | --- | --- | --- | --- | --- | --- | --- | --- | --- | --- | --- |
| Variables | *b* | SE | *p* |  | *b* | SE | *p* |  | *b* | SE | *p* |
| Age  35-54  55 or above | 1.581  0.118 | 0.981  1.081 | 0.108  0.913 |  | -0.121  -2.411 | 0.966  1.064 | 0.901  0.024 |  | -0.314  -1.393 | 0.567  0.624 | 0.580  0.026 |
| Sex  Male | -0.694 | 0.794 | 0.382 |  | 0.874 | 0.780 | 0.264 |  | -0.019 | 0.458 | 0.967 |
| Race  Black/African American  Other | -1.629  -2.490 | 0.885  1.209 | 0.067  0.040 |  | -0.441  0.152 | 0.872  1.189 | 0.613  0.898 |  | -0.676  -0.058 | 0.512  0.698 | 0.187  0.934 |
| Ethnicity  Hispanic  Unknown | 2.574  2.058 | 1.527  2.230 | 0.092  0.357 |  | 0.629  0.158 | 1.502  2.194 | 0.675  0.943 |  | -0.334  -1.022 | 0.881  1.288 | 0.705  0.428 |
| Marital Status  Married  Other | 0.987  1.724 | 1.115  1.243 | 0.377  0.166 |  | 0.201  -0.358 | 1.094  1.233 | 0.854  0.772 |  | -0.455  -0.826 | 0.645  0.728 | 0.480  0.258 |
| Income Level  $20,000-$74,999  $75,000 or more | -0.063  -1.026 | 1.039  1.184 | 0.952  0.387 |  | -2.024  -3.358 | 1.003  1.169 | 0.044  0.004 |  | -1.490  -1.350 | 0.595  0.686 | 0.013  0.050 |
| Pandemic Phase  Phase 2  Phase 3  Phase 4 | -0.065  1.752  5.518 | 1.087  1.104  1.201 | 0.952  0.113  <0.001 |  | 1.857  0.492  -0.189 | 1.069  1.086  1.181 | 0.083  0.651  0.873 |  | 1.636  0.261  0.393 | 0.628  0.638  0.693 | 0.009  0.683  0.571 |
| Low Positive Coping | 2.154 | 1.421 | 0.130 |  | 3.290 | 1.400 | 0.019 |  | 2.678 | 0.822 | 0.001 |
| AUD History | 11.348 | 0.912 | <0.001 |  | 2.143 | 0.896 | 0.017 |  | 1.760 | 0.527 | 0.001 |
| Low Positive Coping x AUD History | 6.200 | 2.082 | 0.003 |  | 2.686 | 2.049 | 0.191 |  | 1.741 | 1.203 | 0.149 |
|  | PHQ-9 (*n* = 463) | | |  | UCLA-LS (*n* = 366) | | |  | DMQ-Coping (*n* = 310) | | |
| Variables | *b* | SE | *p* |  | *b* | SE | *p* |  | *b* | SE | *p* |
| Age  35-54  55 or above | -0.599  -1.795 | 0.610  0.673 | 0.327  0.008 |  | -0.556  -1.510 | 2.110  2.358 | 0.792  0.523 |  | 0.596  -0.104 | 0.635  0.703 | 0.349  0.883 |
| Sex  Male | 0.378 | 0.493 | 0.443 |  | 0.911 | 1.741 | 0.601 |  | 0.157 | 0.587 | 0.790 |
| Race  Black/African American  Other | -0.806  -0.700 | 0.550  0.751 | 0.144  0.352 |  | -4.409  -3.801 | 1.899  2.599 | 0.021  0.145 |  | -0.759  -1.091 | 0.620  0.817 | 0.223  0.184 |
| Ethnicity  Hispanic  Unknown | -0.005  -0.583 | 0.948  1.385 | 0.996  0.674 |  | -1.543  -4.442 | 3.390  4.524 | 0.649  0.327 |  | 0.832  0.553 | 1.005  1.463 | 0.409  0.706 |
| Marital Status  Married  Other | -0.049  -0.091 | 0.697  0.778 | 0.944  0.907 |  | -3.345  -0.513 | 2.506  2.768 | 0.184  0.853 |  | 1.096  1.128 | 0.767  0.841 | 0.155  0.182 |
| Income Level  $20,000-$74,999  $75,000 or more | -0.759  -1.325 | 0.645  0.738 | 0.240  0.074 |  | -2.406  -5.126 | 2.532  2.889 | 0.345  0.080 |  | -0.091  -2.781 | 0.771  0.788 | 0.907  0.001 |
| Pandemic Phase  Phase 2  Phase 3  Phase 4 | 1.453  0.062  0.105 | 0.675  0.686  0.746 | 0.032  0.928  0.888 |  | 1.248  -0.446  -0.729 | 2.448  2.463  2.788 | 0.611  0.856  0.794 |  | -1.581  -1.441  0.193 | 0.790  0.798  0.799 | 0.048  0.074  0.809 |
| Low Positive Coping | 2.803 | 0.883 | 0.002 |  | 2.873 | 3.045 | 0.346 |  | 0.472 | 0.926 | 0.611 |
| AUD History | 2.068 | 0.567 | <0.001 |  | 5.670 | 2.008 | 0.005 |  | 4.777 | 0.668 | <0.001 |
| Low Positive Coping x AUD History | 3.435 | 1.294 | 0.008 |  | 5.313 | 4.493 | 0.238 |  | 3.616 | 1.295 | 0.006 |
