# Supplementary material for: Exercising healthy behaviors: A latent class analysis of positive coping during the COVID-19 pandemic and associations with alcohol-related and mental health outcomes
Source: PLoS One. 2024 Feb 14;19(2):e0297060. doi: 10.1371/journal.pone.0297060 (PMC10866465; doi:10.1371/journal.pone.0297060)
Supplement: Attachment — Submitted filename: referee_report.pdf [file pone.0297060.s002.pdf]

Referee Report: Exercising healthy behaviors: A latent class analysis of positive coping during the COVID-19 pandemic and associations with alcohol-related and mental health outcomes.

This paper utilizes a latent class analysis approach to identify distinct groups of individuals based on their positive coping behaviors and investigates the association between these groups and both alcohol-related and mental health outcomes, while considering the presence or absence of a history of alcohol use disorder (AUD). Employing a variety of coping behavior responses, the results reveal the existence of two latent classes - namely high positive coping and low positive coping. High positive coping behavior is found to be inversely related to negative coping behaviors. The results indicate that individuals with a history of AUD are more likely to experience alcohol-related and mental health problems. Furthermore, low positive coping behaviors, as well as their interaction with a history of AUD, have significant associations with some of these outcomes.

Overall, I found the paper to be informative and interesting but there are a few areas where revision and further clarification could improve the paper.

Comments:

1. The paper includes a large number of analyses, but for some of them it was not clearly communicated in the paper why certain analyses were conducted. For instance, the analysis that looks at the relationship between positive and negative coping behavior in page 13 (Table 3). It is not clear why this analysis was done. Was it a sanity check of the latent classes or did it have additional purpose? For the sake of clarity and completeness, the authors should explain more clearly the purpose and relevance of each analysis to the research question. This would help readers to follow the logic and importance of each analytical step taken.

2. In the statistical analysis, page 9, the authors talk about pandemic phases, but we do not know what time period constitutes these phases unless we read the caption in Table 1. The authors should discuss the pandemic phases in the main text and what data was collected in which phase.

3. The section "Latent Class Differences in Alcohol-Related and Mental Health Outcomes and Moderation by History of AUD" on page 14 requires more detailed discussion of the results by the different outcome variables in Figure 2.

4. Figure 2 requires a caption explaining what the bar represents (mean/ average of the outcomes) and what the error bars represents (e.g., 95% confidence interval)

5. Table 4 is too crowded. Since the other covariates such as age and gender are not discussed in the paper, the authors can exclude those variables from the table and just show the estimates for low positive coping, AUD and their interaction. The full table with all the covariates can be included in the appendix. The authors should also include the sample size for all the outcome variables.

6. In line 313 on page 19, the sentence talks about the findings of this study but provides citations of other papers. I am not sure why that was done.

7. In the discussion section, from page 19 onwards, the authors make more general statements about how positive coping behavior may be protective against alcohol related and mental health outcomes

while the authors findings in the paper were more nuanced. For example, the regression analysis shows that low positive coping is only associated with alcohol related outcomes for individuals with AUD. While the authors' discussion also focuses on the outcomes that they found a significant relationship with, I think the readers will also benefit from discussion of the outcomes that the authors did not see a significant association with. For instance, in line 322 page 19, the authors cite and discusses a study that found how positive coping behaviors could be used to prevent feelings of distress and loneliness, however, in this study we do not see positive coping behavior to be associated with loneliness.

8. Minor comment: On page 5, SCID-IV and SCID-5 use two different numerals in the name. Unless this is how the names should be written, perhaps either use English or Roman numerals.
