# Supplementary material for: Exercising healthy behaviors: A latent class analysis of positive coping during the COVID-19 pandemic and associations with alcohol-related and mental health outcomes
Source: PLoS One. 2024 Feb 14;19(2):e0297060. doi: 10.1371/journal.pone.0297060 (PMC10866465; doi:10.1371/journal.pone.0297060)
Supplement: Attachment — Submitted filename: C19_McCabe_HealthyLCA_PLOS_One_Response to Reviewers.docx [file pone.0297060.s003.docx]

**Responses to Reviewers’ Comments**

**Reviewer #1:** This paper utilizes a latent class analysis approach to identify distinct groups of individuals based on their positive coping behaviors and investigates the association between these groups and both alcohol-related and mental health outcomes, while considering the presence or absence of a history of alcohol use disorder (AUD). Employing a variety of coping behavior responses, the results reveal the existence of two latent classes - namely high positive coping and low positive coping. High positive coping behavior is found to be inversely related to negative coping behaviors. The results indicate that individuals with a history of AUD are more likely to experience alcohol-related and mental health problems. Furthermore, low positive coping behaviors, as well as their interaction with a history of AUD, have significant associations with some of these outcomes.

Overall, I found the paper to be informative and interesting but there are a few areas where revision and further clarification could improve the paper.

Comments:

1. The paper includes a large number of analyses, but for some of them it was not clearly communicated in the paper why certain analyses were conducted. For instance, the analysis that looks at the relationship between positive and negative coping behavior in page 13 (Table 3). It is not clear why this analysis was done. Was it a sanity check of the latent classes or did it have additional purpose? For the sake of clarity and completeness, the authors should explain more clearly the purpose and relevance of each analysis to the research question. This would help readers to follow the logic and importance of each analytical step taken.

**RESPONSE: In the revised manuscript, each of the study goals are laid out in numerical order in the introduction and new literature has been added to support the research questions. Specifically, the negative coping analysis is now listed as the first goal of this manuscript (Page 5, paragraph 2). Existing literature describes how the pandemic has led to increases in negative coping and unhealthy lifestyle behaviors among some individuals. By including the analyses examining the associations between positive and negative coping behaviors, it would help us better connect our findings to the existing literature. We have made edits throughout the introduction. As examples, we have added the following sentences to strengthen our justification of this study goal:**

**“*Individuals with a history of AUD may be more vulnerable during times of high stress as their positive coping skills may be underdeveloped, leading to the adoption of negative coping strategies and unhealthy lifestyle behaviors.*” (Page 3, paragraph 2)**

***“Much of the emerging research around COVID-19 impact has studied the negative health-related consequences of lockdown and upticks in endorsement of negative coping behaviors [8, 23, 25-28]. Pandemic-related stress has been associated with reports of elevated loneliness, mental health problems, and drinking to cope [20-22, 25, 29-31]. Individuals who adopted or maintained a healthier lifestyle during the pandemic were more likely to experience better physical and mental health outcomes [8, 23, 32].”* (Pages 3-4, paragraph 3)**

2. In the statistical analysis, page 9, the authors talk about pandemic phases, but we do not know what time period constitutes these phases unless we read the caption in Table 1. The authors should discuss the pandemic phases in the main text and what data was collected in which phase.

**RESPONSE: Thank you for this suggestion. We have added the time periods for the pandemic phases in the revised Participants section within Methods:**

***“Given rolling enrollment in the C19-PIA Study, participants were enrolled at different stages of the pandemic. Four pandemic phases were created based on frequency distributions of enrollment timing while considering COVID-19 infection level and state policies in the Washington D.C. Metro area: Phase 1 (June 3 to July 31, 2020); Phase 2 (August 1 to November 22, 2020); Phase 3 (November 23, 2020 to February 28, 2021); and Phase 4 (March 1, 2021 to January 6, 2022).”* (Page 6)**

3. The section “Latent Class Differences in Alcohol-Related and Mental Health Outcomes and Moderation by History of AUD” on page 14 requires more detailed discussion of the results by the different outcome variables in Figure 2.

**RESPONSE: We have included a summary of the study findings in the revised results section: *“Fig 2 shows the pattern of results regarding the associations between Positive Coping Latent Classes and outcomes of interest by history of AUD. There were significant interaction effects between Low Positive Coping and history of AUD on alcohol problems (Panel A), depressive symptoms (Panel D), and drinking to cope (Panel F). The overall main effects of Low Positive Coping were statistically significant for perceived stress (Panel B), anxiety symptoms (Panel C), and loneliness (Panel E). The main effects of AUD were statistically significant for all clinical outcomes (Panels A-F).”* (Pages 14-15)**

**Please note that the decomposition of the interaction effects is presented in the next paragraph while referencing S1 Table 1 and Table 4. Together, we have provided more detailed interpretation of the study findings in these two revised paragraphs.**

4. Figure 2 requires a caption explaining what the bar represents (mean/average of the outcomes) and what the error bars represents (e.g., 95% confidence interval)

**RESPONSE: A figure caption was added to Figure 2 including the suggested representations: “*The blue and orange bars indicate the total or mean score for each outcome measure. The black error bars depict 95% confidence intervals (CIs). All pairwise comparisons were significant at the p < 0.05 level, except for the difference between non-AUD high positive coping and non-AUD low positive coping groups (Panel A, E, F), and the difference between non-AUD low positive coping and AUD high positive coping groups (Panels B, C, D, E).”* (Page 15)**

5. Table 4 is too crowded. Since the other covariates such as age and gender are not discussed in the paper, the authors can exclude those variables from the table and just show the estimates for low positive coping, AUD and their interaction. The full table with all the covariates can be included in the appendix. The authors should also include the sample size for all the outcome variables.

**RESPONSE: The recommended changes were made so that only main and interaction effects are included in the revised Table 4. Sample sizes for each outcome were addressed in the table caption, as were adjustments for multiple imputation (Page 16). The full table with all the covariates has been included as S1 Table in the appendix.**

6. In line 313 on page 19, the sentence talks about the findings of this study but provides citations of other papers. I am not sure why that was done.

**RESPONSE: We have removed the sentence in question from the revised manuscript and have reserved citations only as supporting evidence for our study findings within the discussion.**

7. In the discussion section, from page 19 onwards, the authors make more general statements about how positive coping behavior may be protective against alcohol related and mental health outcomes, while the authors findings in the paper were more nuanced. For example, the regression analysis shows that low positive coping is only associated with alcohol related outcomes for individuals with AUD. While the authors’ discussion also focuses on the outcomes that they found a significant relationship with, I think the readers will also benefit from discussion of the outcomes that the authors did not see a significant association with. For instance, in line 322 page 19, the authors cite and discuss a study that found how positive coping behaviors could be used to prevent feelings of distress and loneliness, however, in this study we do not see positive coping behavior to be associated with loneliness.

**RESPONSE: Thank you for this helpful feedback. We have significantly changed and reorganized our discussion accordingly. First, the fifth and sixth paragraphs in the initial draft (those presented from page 19 onwards) were removed. Second, we added a new paragraph as the third paragraph that focuses on the interpretation on the interaction effects for three of the outcomes - problematic drinking, depressive symptoms, and drinking to cope. Third, we revised the fourth paragraph and refocused it on the main effects of positive coping on perceived stress, anxiety symptoms, and loneliness. Finally, we added another new paragraph that discusses several covariate effects. We believe the reorganized discussion has removed some of the problematic text (e.g., general statements that did not sufficiently distinguished interaction findings vs. main effects, or the problematic citation) and is more effective in highlighting the nuances in the current study findings.**

8. Minor comment: On page 5, SCID-IV and SCID-5 use two different numerals in the name. Unless this is how the names should be written, perhaps either use English or Roman numerals.

**RESPONSE: The SCID is based on the DSM, and the DSM versions are officially written as “SCID-IV” and “SCID-5,” respectively. To clarify this point, we have used the full name of these two versions instead of the abbreviations: *“Based on data from Structured Clinical Interview for DSM-IV or DSM-5 collected as part of the NHP, 41.9% (n = 194) of participants met criteria for an AUD diagnosis.”* (Page 6)**

**Reviewer #2:** In this cross-sectional study, authors identify latent classes of coping behaviors during the first year of the COVID-19 pandemic and examine associations with alcohol-related and mental health outcomes among participants with and without alcohol use disorder (AUD). The study used data collected from a subset of participants in an existing NIAAA cohort study and data was collected online and by phone over the first year of the pandemic. Latent class analysis, chi-squares, and regression models were used to analyze results. Results showed that participants with low positive coping had significantly greater alcohol use and poorer mental health outcomes and were more likely to endorse negative coping behaviors.

The manuscript is written clearly and is well-supported by literature. There are a few additional topics to consider for inclusion in the discussion:

It’s unclear to me why the author focuses on “more likely to report being single or married as opposed to ‘other.’” Single versus married seems to be a much more disparate distinction than “other” and there’s no literature to support why marital status may be playing a role in latent classes or behaviors.

**RESPONSE: We have removed the problematic text in the original draft and have clarified it as: *“Chi-square tests run on marital status and pandemic phase by latent class were statistically significant, suggesting that positive coping varied by marital status and pandemic phase. Specifically, compared to participants with High Positive Coping, participants with Low Positive Coping were less likely to be “married” (11.7% vs 22.7%) and more likely to report “other” marital status (e.g., divorced, separated, widowed; 23.4% vs 12.3%).”* (Page 10)**

**We have also included a discussion point with newly identified citations:**

***“In our study, we found that low positive coping was more frequently endorsed by individuals who were divorced, separated, or widowed. This group of individuals may lack support from their family to engage in positive coping, or they may have less free time or lower financial resources to support certain types of positive coping (e.g., exercising regularly or making time to relax) [68-70]. Community-based support groups for these individuals may help encourage positive coping behaviors [71].”* (Page 18)**

There is no discussion of how pandemic phase may play a role in results, although it is highlighted as a participant characteristic in Table 1. We know that recommended behavioral guidelines shifted quite a bit throughout these phases, as did the perceived severity of outbreak as different variants were introduced. Seems like phase would be an important thing to consider when assessing when and why people were employing specific coping strategies and the paper would be significantly strengthened by adding some sort of discussion and literature around this consideration.

**RESPONSE: We have added the corresponding text in the revised results section: *“Low Positive Coping was more frequently endorsed during the early phase of the pandemic (Phase 1, 37.7%), whereas High Positive Coping was more frequently endorsed from August 1 to November 22, 2020 (Phase 2, 32.6%).”* (Page 10)**

**One interpretation could be that participants generally needed time to adjust to the pandemic initially, and thus after the initial adjustment period, they were able to cope better in the second phase and beyond. However, as this interpretation is somewhat speculative, we have expanded the issue of pandemic phase as a potential confounder in the study limitation: “Fifth, although pandemic phases were controlled for as a covariate in the analyses, shifts in pandemic-related mandates related to quarantining and social distancing may have influenced levels of stress, time availability, and motivation to engage in positive or negative coping behaviors, as well as the assessed outcomes [24, 64, 77-78].” (Page 19)**

While the mention of future directions investigating predictive factors that prime individuals to select coping behaviors is interesting and well cited, suggest that the authors tie this back to their own work and how the demographics of their own sample may or may not reflect the potential predictors they reference in this section of the discussion. How does the makeup of your sample support or not support these findings?

**RESPONSE: In general, our study sample was quite diverse. We have highlighted this point in the revised results section: *“The study sample had a mean age of 44.8 (SD = 14.2) and was diverse in terms of sex, race, and ethnicity.”* (Page 10)**

**After reviewing the paragraph in the discussion, we recognized that the narrative was disjointed and did not connect back to our study findings. Therefore, we have rewritten this paragraph entirely so that the discussion is more closely tied to the study findings. The new paragraph now reads:**

**“*To best inform targeted dissemination of positive coping strategies, it is important to understand whether segments of our society may be less likely to utilize positive coping. In our study, we found that low positive coping was more frequently endorsed by individuals who were divorced, separated, or widowed. This group of individuals may lack support from their family to engage in positive coping, or they may have less free time or lower financial resources to support certain types of positive coping (e.g., exercising regularly or making time to relax) [68-70]. Community-based support groups for these individuals may help encourage positive coping behaviors [71]. Prior literature also suggests that individuals with higher socioeconomic status may have greater access to resources like money, time, and social support to promote healthy lifestyle behaviors [29, 72-73]. Although we did not find significant differences in income level by latent classes, in theory, individuals with greater financial resources would have access to more positive coping activities (e.g., joining a gym membership or buying healthier food). More research is needed to examine how financial and social capital can be used to help promote positive coping behaviors.*” (Pages 18-19)**

Minor Suggestions:
Relatively minor changes to the organization of the introduction would strengthen its argument. Though all the information is there and well cited, it feels a bit disjointed. Suggest reorganizing the section to discuss general research on the effect of AUD, health behaviors, and stress on the population and then shift to how the pandemic may have exacerbated existing associations rather than switching back and forth between general and pandemic related findings to more clearly delineate arguments

**RESPONSE: The introduction was rearranged accordingly to first discuss the influence of health behaviors and substance use in the general population and how those are influenced by stress (Page 3, paragraph 1). Next, alcohol use disorder (AUD) as a public health issue and vulnerability in stress and coping among individuals with AUD were introduced (Page 3, paragraph 2). Then, the next two paragraphs transitioned to discuss the COVID-19 pandemic as a stressful event that may have exacerbated the negative health consequences and prior literature on lifestyle coping behaviors during the pandemic were reviewed (Pages 3-4). We believe that this reorganization has reduced the back and forth** **between general and pandemic related findings and the revised narrative is much more focused and coherent.**

Methods - The author states “Participants included healthy volunteers, moderate social drinkers, and individuals with alcohol use disorder (AUD).” It’s unclear what these categories mean at this point and would suggest deleting this sentence or moving it to later in the methods section when it is clearer what they are referring to.

**RESPONSE: We have followed the Reviewer’s recommendation and have deleted this sentence.**

Results - Please correct formatting in the frequency box of the Pandemic Phase section of Table 1. Interesting that there is a clear difference in high and low-coping behaviors during Phases 1 and 2 of the pandemic, but I don’t see that mentioned in the initial results.

**RESPONSE: We have corrected the formatting of Table 1. Marital status and pandemic phase findings from Table 1 are now mentioned at the beginning of the Results, under Sample Characteristics: *“Most sample characteristics did not significantly differ between latent classes. Chi-square tests run on marital status and pandemic phase by latent class were statistically significant, suggesting that positive coping varied by marital status and pandemic phase. Specifically, compared to participants with High Positive Coping, participants with Low Positive Coping were less likely to be “married” (11.7% vs 22.7%) and more likely to report “other” marital status (e.g., divorced, separated, widowed; 23.4% vs 12.3%). Low Positive Coping was more frequently endorsed during the early phase of the pandemic (Phase 1, 37.7%), whereas High Positive Coping was more frequently endorsed from August 1 to November 22, 2020 (Phase 2, 32.6%).”* (Page 10)**

**Reviewer #3:** This manuscript reports an analysis of data from a cross-sectional survey of 463 adults, about half of whom had alcohol use disorder (AUD). Surveys were completed between June 2020 and January 2022, during the COVID-19 pandemic. The authors fit a latent class model to self-reported data on positive coping behaviors, then related class membership to negative coping behaviors, problematic alcohol use, perceived stress, anxiety, depression, loneliness, and drinking to cope.
The authors chose a two-class latent class solution and found that relative to the “high positive coping” class, the “low positive coping” class exhibited more negative coping behaviors, perceived stress, anxiety, and depression. Moderation analyses showed stronger associations between membership in the high vs. low positive coping classes and outcomes for those with history of AUD.
I believe more information is needed about the design and analysis before the manuscript could be described as technically sound.

BIGGEST ISSUES
1. Why was a latent class model used? The few sentences lines 84-91 are mostly an assertion that the LCA model is valuable rather than an explanation of why that is so. An LCA is a complex model and the choice of this model is not much discussed or justified relative to more straightforward approaches. You could have tested how each positive coping behavior related to each outcome (and, e.g., made an adjustment for multiple testing). Why didn’t you do that? You could then have issued more specific recommendations about which positive coping behaviors have which effects.

**RESPONSE: Positive coping behaviors tend to co-occur within person*.* While it is true that pairwise analyses can be conducted with an adjustment for multiple testing, we find the latent class model to be more integrative and clinically meaningful. In the literature, LCA is often cited as a superior approach when the research question involves identifying subgroups of individuals that share various characteristics, and in this case, the characteristics of interest are different types of positive coping behaviors. As a person-centered approach, LCA effectively helps us understand the proportion of the study sample with high versus low positive coping behaviors. If we utilized a variable-centered approach, we would not be able to discover that low utilization of positive coping strategies is primarily clustered in a small group of individuals (16.6%). Overall, the identification of latent classes can inform targeted clinical interventions. That is, instead of promoting any specific positive coping behavior for everyone, our findings suggest that the 77 participants (16.6%) with low positive coping would likely benefit from coping skills training, in which multiple options for positive coping can be taught simultaneously.**

**To strengthen our rationale for using LCA, we have now devoted a full paragraph to clearly state three specific reasons why we think the application of LCA is suitable for our research questions in this study:**

***“Latent class analysis (LCA) is a person-centered approach that has been utilized to examine distinct patterns of COVID-related stressors and experiences, including work-related interruptions, changes to home life, experiences of social isolation, and financial stress [22, 38-39]. There are several advantages to this analytic approach [40-41]. First, LCA is an empirical approach that identifies subgroups of individuals who share similar characteristics based on an underlying latent construct, such as COVID-related stress or COVID-related coping. The application of LCA can help researchers understand the degree to which different types of stressors or coping strategies co-occur among individuals and allow for the opportunity to examine their cumulative effects on external variables. Second, the identification of subgroups of individuals who are at varying levels of risk for mental health and alcohol-related problems can inform targeted outreach and intervention among those most affected. Third, LCA can help integrate information from several observable indicators and capture patterns of endorsement effectively. As a result, more integrated analyses can be conducted using the latent classes rather than the individual indicators. Despite these advantages, LCA has not been applied to examine positive coping behaviors in the context of the COVID-19 pandemic.”* (Pages 4-5)**

2. I think the manuscript should better address the question of reverse causality. You write about positive coping having effects on negative forms of coping, how do we know it is not the opposite? You write about positive coping reducing risk of mental health and substance use outcomes, how do we know it is not the opposite? Can you rule this out in any way and if not, how does it qualify your findings? You briefly acknowledge this possibility in lines 341-342 but the rest of the Discussion and the Abstract is written as if direction of causality has been established.

**RESPONSE: Thank you for this feedback. We have reviewed the paper and the use of causal language (e.g., impact, effect, etc.) was avoided whenever feasible. For instance, in the abstract, we have altered the language of the sentence from *“Significant interactions between Coping Latent Classes and history of AUD indicated that the negative impact of Low Positive Coping on problematic alcohol use, depressive symptoms, and drinking to cope motives were either stronger or only significant among individuals with a history of AUD.”* to *“Significant interactions between Coping Latent Classes and history of AUD indicated that the associations of Low Positive Coping with problematic alcohol use, depressive symptoms, and drinking to cope motives were either stronger or only significant among individuals with a history of AUD.”* (Page 2)**

**Moreover, the study limitation about the use of cross-sectional data was expanded with the new addition of a specific example: *“First, the cross-sectional design prohibited causal interpretation. For example, while positive coping strategies can be used to manage symptoms of anxiety and depression, anxiety and depression can also lead to lower use of positive coping as individuals with anxiety or depression may be more prone to avoidant behavior or social isolation [74-76]. Longitudinal research is needed to disentangle the directionality of effects.”* (Page 19).**

OTHER ISSUES
3. I think you should provide at least a brief characterization of sample recruitment in this manuscript. I do not think writing “Details about the study design and participant recruitment procedures can be found elsewhere” (line 112) is sufficient.

**RESPONSE: We have included a description of the study recruitment and procedures in the revised Methods/Participants section:**

**“*In brief, individuals who previously participated in research at NIAAA via the Natural History Protocol (NHP) were invited to join a study focused on the impact of the pandemic on alcohol-related behaviors [43]. Participants provided verbal consent at the start of the initial phone survey and consent was documented as part of the survey. Participants were provided a copy of the consent document along with their initial online survey. The baseline survey consisted of two parts, with the first part conducted over the phone, and the second part conducted either over the phone or online. Participants who completed the first part were compensated with $25, and those who completed both parts were compensated with $50.”* (Pages 5-6)**

4. Please report the centering of variables in the product term for models with interaction (Table 4), since this affects interpretation.

**RESPONSE: Thank you for this suggestion. We have clarified that the product term was created after centering. A sentence has been added to the revised Statistical Analysis section: *“The interaction term was created using centered Positive Coping Latent Classes (0 = High Positive Coping and 1 = Low Positive Coping) and centered History of AUD (0 = no history of AUD; 1 = history of AUD).”* (Page 9)**

5. Please report basic information about how you did the multiple imputation (e.g., number of imputations, specification of the chained equations).

**RESPONSE: More information has been provided for multiple imputation procedures in the revised Statistical Analysis section: *“The number of imputations was 20 and specification of chained equations was performed based on characteristics of the imputed variable (e.g., ordered logistic regression for income, and linear regressions for continuous variables).”* (Page 10)**

6. Lines 270-273… should “drinking-to-cope motives” actually be on this list of significant differences between the latent classes? My reading of tables/figures is that they did not significantly differ on DMQ-coping.

**RESPONSE: We have deleted the problematic sentence identified by the Reviewer. The sentence in the initial draft was problematic because it did not differentiate between the three clinical outcomes where there were only main effects of positive coping and AUD versus the other three clinical outcomes where the associations between positive coping and the clinical outcomes were conditioned by history of AUD. This confusion has now been clarified in the revised discussion.**

7. FIGURE 2. Please add a note/legend. What are the error bars indicating, +/- 2 SEs? Can you indicate here which contrasts are significant? The error bars around each mean do not answer that question.

**RESPONSE: Thank you for this excellent recommendation. We have clarified these critical issues in the Figure caption:
*“Note: The blue and orange bars indicate the total or mean score for each outcome measure. The black error bars depict 95% confidence intervals (CIs). All pairwise comparisons were significant at the p < 0.05 level, except for the difference between non-AUD high positive coping and non-AUD low positive coping groups (Panel A, E, F), and the difference between non-AUD low positive coping and AUD high positive coping groups (Panels B, C, D, E).”* (Page 15)**

8. Did you test any interactions with the pandemic phase? Your observations cover a pretty large variety of pandemic contexts, from June 2020 to January 2022.

**RESPONSE: We did not test interactions with the pandemic phase since we were mostly interested in the interactions between Positive Coping and history of AUD on clinical outcomes. We included pandemic phase as a study covariate, which serves as a proxy for contextual and policy changes throughout the pandemic. The full regression models presented in S1 Table show all the regression coefficients for the study covariates, including pandemic phase. To further address the Reviewer’s concern, we have also expanded upon the study limitation regarding the potential confounding of pandemic phase:**

***“Fifth, although pandemic phases were controlled for as a covariate in the analyses, shifts in pandemic-related mandates related to quarantining and social distancing may have influenced levels of stress, time availability, and motivation to engage in positive or negative coping behaviors, as well as the assessed outcomes [24, 64, 77-78].”* (Page 19)**

**We hope these changes to the manuscript have sufficiently addressed the Reviewers’ concerns. Once again, we thank the Reviewers for their valuable time and constructive feedback which has helped us improve the quality of our work.**
